# Dysregulated expression of proteins associated with ER stress, autophagy and apoptosis in tissues from nonalcoholic fatty liver disease

**DOI:** 10.18632/oncotarget.18812

**Published:** 2017-06-28

**Authors:** Seungwoo Lee, Soohee Kim, Seungwoo Hwang, Nathan J. Cherrington, Doug-Young Ryu

**Affiliations:** ^1^ BK21 Plus Program for Creative Veterinary Science Research, Research Institute for Veterinary Science and College of Veterinary Medicine, Seoul National University, Gwanak-gu, Seoul 08826, Republic of Korea; ^2^ Korean Bioinformation Center, Korea Research Institute of Bioscience and Biotechnology, Daejeon 34141, Republic of Korea; ^3^ College of Pharmacy, University of Arizona, Tucson, AZ 85721, U.S.A

**Keywords:** nonalcoholic fatty liver disease, endoplasmic reticulum stress, apoptosis, autophagy

## Abstract

Nonalcoholic fatty liver disease (NAFLD) is categorized into nonalcoholic fatty liver (NAFL) and nonalcoholic steatohepatitis (NASH) and has emerged as a risk factor for more critical clinical conditions. However, the underlying mechanisms of NAFLD pathogenesis are not fully understood. In this study, expression of proteins associated with endoplasmic reticulum (ER) stress, apoptosis and autophagy were analyzed in normal, NAFL and NASH human livers by western blotting. Levels of some ER stress-transducing transcription factors, including cleaved activating transcription factor 6, were higher in NASH than in the normal tissues. However, the expression of a majority of the ER chaperones and foldases analyzed, including glucose-regulated protein 78 and ER protein 44, was lower in NASH than in the normal tissues. Levels of apoptosis markers, such as cleaved poly (ADP-ribose) polymerase, were also lower in NASH tissues, in which expression of some B-cell lymphoma-2 family proteins was up- or down-regulated compared to the normal tissues. The level of the autophagy substrate p62 was not different in NASH and normal tissues, although some autophagy regulators were up- or down-regulated in the NASH tissues compared to the normal tissues. Levels of most of the proteins analyzed in NAFL tissues were either similar to those in one of the other two types, NASH and normal, or were somewhere in between. Together, these findings suggest that regulation of certain important tissues processes involved in protein quality control and cell survival were broadly compromised in the NAFLD tissues.

## INTRODUCTION

Nonalcoholic fatty liver disease (NAFLD) is a pathological condition histologically categorized into nonalcoholic fatty liver (NAFL) and nonalcoholic steatohepatitis (NASH) [1]. NAFLD can progress to cirrhosis and end-stage liver diseases such as hepatocellular carcinoma [2, 3].

Accumulation of unfolded proteins in the endoplasmic reticulum (ER) causes ER stress, which triggers an adaptive response called the unfolded protein response (UPR) to restore ER homeostasis [4]. The UPR pathway is also required to maintain hepatic lipid metabolism [5]. The UPR is coordinated primarily by three ER transmembrane stress transducers, protein kinase RNA-like ER kinase (PERK), activating transcription factor 6 (ATF6) and inositol requiring enzyme 1 (IRE1).

Prolonged ER stress leads to PERK signaling-mediated upregulation of C/EBP homologous protein (CHOP), a pro-apoptotic transcription factor [6, 7]. One mechanism by which CHOP induces apoptosis is via inhibition of B-cell lymphoma-2 (Bcl-2) expression and induction of bcl-2-like protein 11 (Bim) expression [8, 9]. ATF6 is a membrane-bound transcription factor, but it is cleaved to release its cytoplasmic domain in response to ER stress. Cleaved ATF6 transcriptionally activates X-box–binding protein 1 (XBP1). XBP1 mRNA is spliced by IRE1 during ER stress to produce the transcription factor, XBP1s [4]. These ER stress-related transcription factors are involved in activation of various ER chaperones and folding-related proteins that directly execute protein quality control [10, 11].

Glucose-regulated protein 78 (GRP78, also known as BiP) and glucose-regulated protein 94 (GRP94) are molecular chaperones regulating protein quality control and degradation. GRP78 also plays a pivotal role in activation of the UPR, in which GRP78 is released from PERK, IRE1 and ATF6 and activates them. The lectin calnexin is a transmembrane ER chaperone involved in folding of newly synthesized glycoproteins [12].

Protein disulfide isomerase (PDI) is a member of PDI superfamily that is involved in oxidative protein folding [13]. ER protein 72 (ERp72) and ER protein 44 (ERp44) are also oxidoreductases in the ER belonging to the PDI family [14]. ER oxireductin 1 (Ero1)-Lα is an oxidase activated during UPR [15, 16].

The intrinsic pathway to apoptosis predominantly leads to cytochrome *c* release from the mitochondria into the cytosol. The intrinsic pathway is strictly controlled by anti-apoptotic (Bcl-2 and myeloid cell leukemia-1 (Mcl-1)) and pro-apoptotic (such as Bim and bcl-2 homologous antagonist/killer (Bak)) Bcl-2 family proteases. The extrinsic pathway to apoptosis can bypass the mitochondrial step. The intrinsic and extrinsic pathways activate caspase-3 protease, which is central to execution of apoptosis [17]. Poly (ADP-ribose) polymerase (PARP) is a cellular substrate of caspases. Cleavage of PARP is considered to be an apoptosis marker [18].

Autophagy is an intracellular pathway responsible for turnover of long-lived proteins [19]. Beclin-1 regulates autophagy, forming a multiprotein complex that initiates autophagosome formation [20]. Autophagy protein 16L1 (Atg16L1) mediates conjugation between autophagy protein 5 (Atg5) and autophagy protein 12 (Atg12) and delivers this complex to autophagosomes. Atg5–Atg12 conjugates convert the cytoplasmic form of microtubule-associated proteins 1A/1B light chain 3A (LC3A/B-I) to the membrane-bound form, referred to as LC3A/B-II. The conversion of LC3A/B-I to LC3A/B-II is a pivotal process for maturation of autophagosomes, enabling their fusion with lysosomes and autophagosome cargo degradation [21]. The protein p62 is a selective substrate for autophagy [19, 22].

Recent evidence suggests the involvement of ER stress and the UPR in development of many chronic liver diseases such as NAFLD [23–28]. Inadequate response to ER stress may cause fat accumulation, insulin resistance, inflammation, autophagy and apoptosis, all of which are critical to pathogenesis of NAFLD [29, 30]. In our study, expression of various proteins associated with ER stress, autophagy and apoptosis was analyzed in NAFL and NASH tissues to elucidate the roles of those proteins in pathogenesis of the critical metabolic disorder.

## RESULTS

### Enhanced expression of transcription factors associated with ER stress in NASH tissues

**Figure 1:**
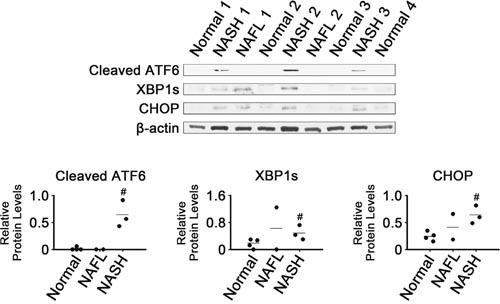
Expression of ER stress-associated transcription factors in NASH, NAFL and normal liver tissues β-Actin was used as an internal control. Horizontal lines represent means of densitometry signals from the western blot analyses for all tissue groups. #, significant differences in signals between NASH and normal liver tissues (*P* < 0.05). Data for NAFL tissues were not used for statistical comparisons because of limited sample number (n = 2).

Expression of three ER stress-responsive transcription factors was analyzed in NASH, NAFL and normal liver tissues by western blotting (Figure 1). Levels of cleaved ATF6, XBP1s and CHOP were higher in NASH than in normal tissues (*P* < 0.05), suggesting that there was activation of these main UPR transducers in NASH. Levels of cleaved ATF6 appeared to be very low in normal and NAFL tissues. It appeared that CHOP levels in NAFL tissues were between those in NASH and normal tissues. The two NAFL tissues displayed highly variable levels of XBP1s and CHOP.

### Decreased expression of ER chaperones in NASH tissues

**Figure 2:**
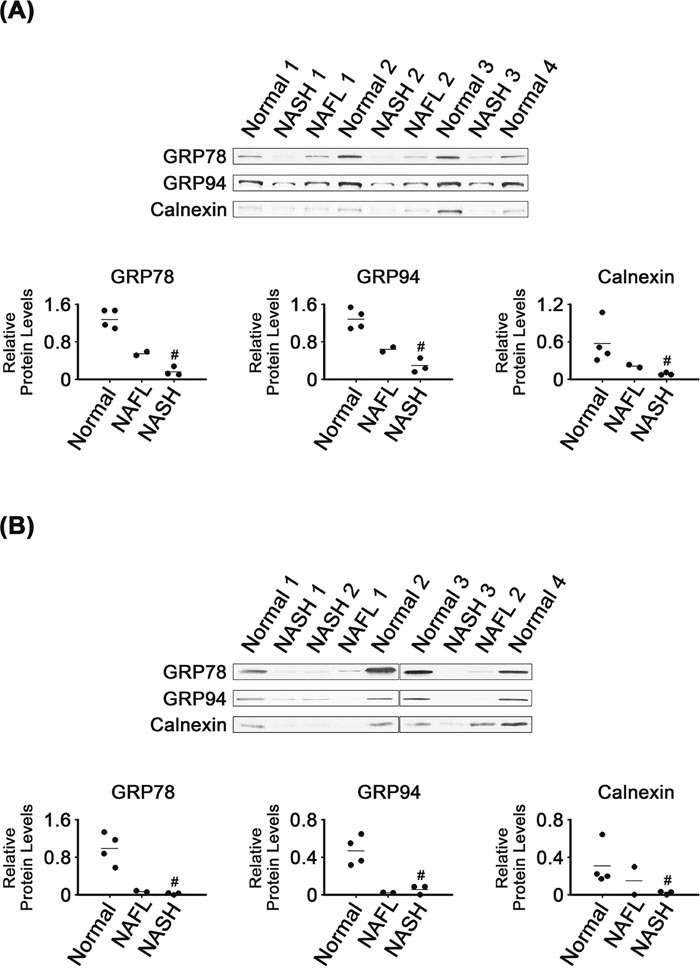
Expression of ER chaperones in NASH, NAFL and normal liver tissues **(A)** and in their microsomes **(B)**. β-Actin was used as an internal control for the whole tissue samples, as described for Figure 1. Horizontal lines represent means of densitometry signals from the western blot analyses for the sample groups. #, significant differences in signals between NASH and normal liver tissues or microsomes (*P* < 0.05). Data for NAFL tissues were not used for statistical comparisons because of limited sample number (n = 2).

In contrast to the enhanced expression of ER stress-associated transcription factors, levels of some ER chaperones were decreased in NASH tissues (Figure 2). Levels of GRP78 and GRP94 were much lower in NASH than in normal tissues (*P* < 0.05; Figure 2A). As found in the whole tissues, the NASH microsomes had lower levels of GRP78 and GRP94 than did normal microsomes (*P* < 0.05; Figure 2B).

It appeared that levels of GRP78 and GRP94 in the NAFL tissues were between those in NASH and normal tissues. The microsomal levels of the two chaperones in NAFL tissues were as low as in NASH microsomes.

The NASH tissues and their microsomes also had lower calnexin levels, as compared with normal tissues and their microsomes, respectively (*P* < 0.05; Figure 2A and 2B). Calnexin levels in the NAFL tissues appeared to be between those in normal and NASH tissues. Levels of calnexin in NAFL microsomes also tended to be between those in normal and NASH microsomes.

### Dysregulated expression of protein foldases in NASH tissues

**Figure 3:**
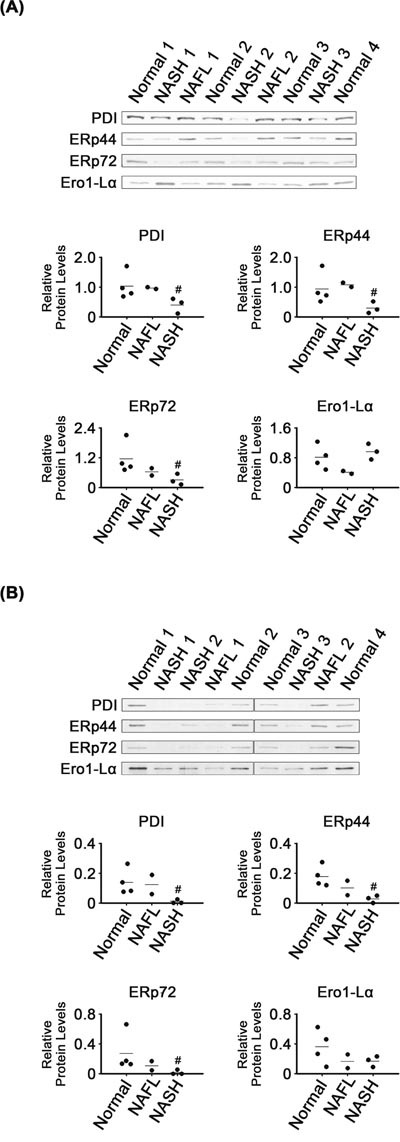
Expression of ER-related foldases in NASH, NAFL and normal liver tissues **(A)** and in their microsomes **(B)**. β-Actin was used as an internal control for the whole tissue samples, as described for Figure 1. Horizontal lines represent means of densitometry signals from the western blot analyses. #, significant differences in signals between NASH and normal liver tissues or microsomes (*P* < 0.05). Data for NAFL tissues were not used for statistical comparisons because of limited sample number (n = 2).

Levels of some enzymes related to protein folding were lower in NASH than in normal tissues. There were lower levels of ER foldases such as PDI, ERp44 and ERp72 in NASH tissues and their microsomes, as compared with in normal tissues and their microsomes, respectively (*P* < 0.05; Figure 3). It appeared that levels of the three foldases in NAFL tissues and microsomes were between those in normal and NASH samples. However, Ero1-Lα levels were not different among the groups, in either tissues or their microsomes.

### Reduced expression of apoptosis markers in NASH tissues

**Figure 4:**
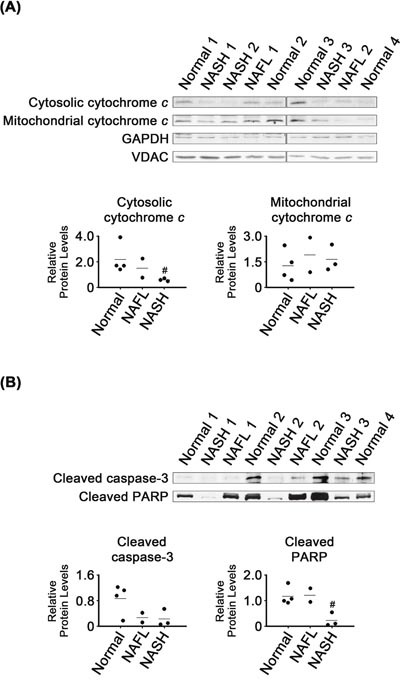
Expression of apoptosis marker proteins in NASH, NAFL and normal liver tissues **(A and B)**. Glyceraldeyde-3-phosphate dehydrogease (GAPDH) and voltage-dependent anion-selective channel (VDAC) were used as internal controls for cytosolic and mitochondrial samples, respectively (A). β-Actin was used as an internal control for the whole tissue samples (B), as described for Figure 1. Horizontal lines represent means of densitometry signals from western blot analyses. #, significant differences in signals between NASH and normal liver tissues (*P* < 0.05). Data for NAFL tissues were not used for statistical comparisons because of limited sample number (n = 2).

Cytosolic cytochrome *c* levels were lower in NASH than in normal tissues (*P* < 0.05; Figure 4A), whereas there were no clear differences in cytochrome *c* levels in mitochondria from these two tissue groups. There were also no significant differences in cleaved caspase-3 levels in normal and NASH tissues (Figure 4B). However, cleaved PARP levels were lower in NASH than in normal tissues (*P* < 0.05). It appeared that cleaved PARP levels in NAFL tissues were as high as in the normal tissues.

### Dysregulated expression of Bcl-2 family proteins in NASH tissues

**Figure 5:**
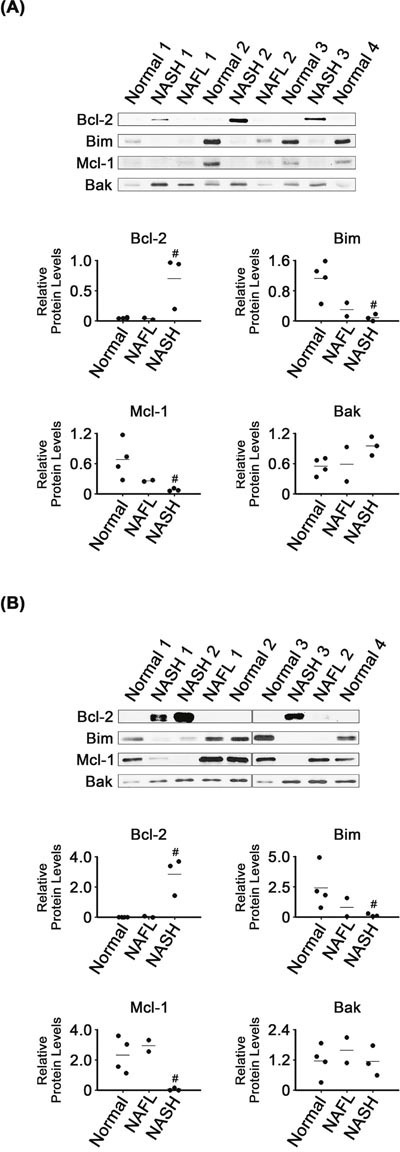
Expression of Bcl-2 family proteins in NASH, NAFL and normal liver tissues **(A)** and in their mitochondria **(B)**. β-Actin and VDAC were used as internal controls for the whole tissue and mitochondria samples, as described for Figures 1 and 4, respectively. Horizontal lines represent means of densitometry signals from the western blot analyses. #, significant differences in signals between NASH and normal liver tissues (*P* < 0.05). Data for NAFL tissues were not used for statistical comparisons because of limited sample number (n = 2).

Bcl-2 levels were much higher in NASH than in normal liver tissues (*P* < 0.05; Figure 5A). Bcl-2 levels in NAFL were as low as in the normal tissues. NASH mitochondria also had higher Bcl-2 levels than those of normal (*P* < 0.05) and NAFL tissues (Figure 5B). Bim levels were lower in NASH tissues and their mitochondria than in the corresponding normal samples (*P* < 0.05). It appeared that expression of the two CHOP-regulated Bcl-2 family proteins was regulated, in NASH tissues, in a manner promoting cell survival.

In contrast to the enhanced expression of Bcl-2, that of Mcl-1, another anti-apoptotic protein, was decreased in NASH tissues and their mitochondria, as compared with in corresponding normal samples (*P* < 0.05). However, it was unclear whether Bak levels were different in NASH and normal tissues or mitochondria.

### Dysregulated expression of autophagy-related proteins

**Figure 6:**
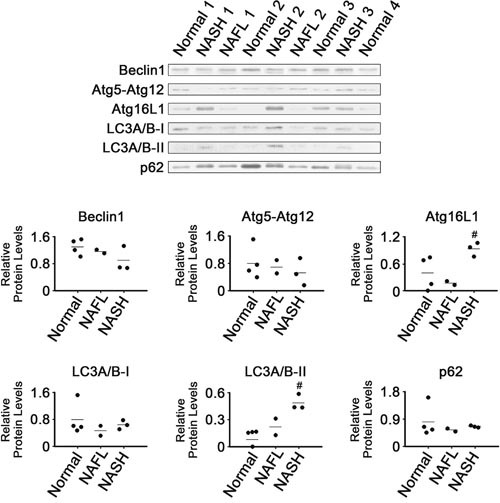
Expression of proteins related to autophagy in NASH, NAFL and normal liver tissues β-Actin was used as internal control, as described for Figure 1. Horizontal lines represent means of densitometry signals from western blot analyses. #, significant differences in signals between NASH and normal liver tissues (*P* < 0.05). Data for NAFL tissues were not used for statistical comparisons because of limited sample number (n = 2).

Expression of autophagy-related proteins was also analyzed (Figure 6). The NASH tissues had higher levels of Atg16L1 and LC3A/B-II than the normal tissues (*P* < 0.05). It appeared that LC3A/B-II levels in NAFL tissues were between those in NASH and normal tissues. However, there were no clear differences in levels of Beclin1, Atg5-Atg12 conjugate or LC3A/B-I among all tissue groups analyzed. In addition, it appeared that levels of p62 were not different among all tissue groups.

## DISCUSSION

XBP1s and cleaved ATF6 are among the major UPR transducers [31]. Thus, the enhanced expression of XBP1s and cleaved ATF6 in the NASH tissues (Figure 1) suggests that ER stress could be sensed within inflammatory tissues, resulting in UPR activation to restore cellular homeostasis. Induction of XBP1s and cleaved ATF6 was also observed in mouse models of NASH [32, 33].

Considering that GRP78 expression was increased in mouse NASH tissues [32, 34] as well as in hepatoma cells treated with palmitate [35], our observation of decreased GRP78 in NASH tissues (Figure 2) was unexpected. It was reported that the expression level of GRP78 mRNA was lower in human NAFL and NASH tissues than in normal liver tissues [27]. Notably, GRP78 downregulation occurred in tissues where expression of XBP1s and cleaved ATF6, two transcriptional regulators for GRP78, were induced (Figure 1) [36–38]. Decreased expression of GRP78 was reported in liver tissues of obese db/db mice, in which expression of cleaved ATF6 was enhanced [35].

Inhibition of GRP78 expression may cause fat accumulation in livers of mice. These mice exhibited increased GRP94 levels, PDI, CHOP, XBP1s and cleaved ATF6 [39]. GRP78 overexpression in the livers of obese ob/ob mice decreased hepatic TG and cholesterol content as well as hepatic expression of XBP1s and ATF6 [40]. Based on these findings, we assumed that decreased GRP78 levels (Figure 2) contributed to the disturbances related to NAFLD and induction of ER stress in liver tissues.

GRP94 depletion did not induce ER stress in the mouse liver, but led to high plasma low-density lipoprotein cholesterol levels [41] as well as to hyperproliferation of mouse liver progenitor cells [42]. In light of these findings, it is possible that GRP94 downregulation in NASH tissues (Figure 2) is involved in dysfunctional lipid metabolism as well as NASH-related proliferative diseases.

Like those of GRP78, GRP94 and calnexin, levels of the ER-associated protein foldases, PDI, ERp44 and ERp72, were also decreased in NASH tissues (Figure 3). Downregulation of the three foldases was unexpected, based on reports that XBP1s could regulate activation of ERp44 and PDI and that ATF6 could, similarly, activate ERp72 [5, 36, 43].

It was intriguing that levels of most of the ER-related chaperones and foldases analyzed in our study were decreased in NASH tissues (Figures 2 and 3), despite activation of the UPR transducers (Figure 1). Because chaperones and foldases are considered to directly execute protein quality control in the ER, it is possible that capacity for protein quality control was broadly compromised in the ER of NASH tissues.

When ER stress cannot be reversed, the UPR can trigger different pathways leading to cell death, such as apoptosis [44]. However, in our study, levels of two apoptosis markers, cytosolic cytochrome *c* and cleaved PARP, were decreased in NASH tissues (Figure 4), suggesting that apoptotic processes were less active than in normal liver. Apoptosis inhibition does not transform cells. However, when it is combined with activation of growth stimulatory signals, cancers can develop [45].

Activated CHOP can cause changes in gene expression favoring apoptosis, including increased Bim and decreased Bcl-2 expression [9, 46]. In contrast to those previously reported observations, CHOP induction (Figure 1) coincided with that of Bcl-2 as well as with inhibition of Bim expression in the NASH tissues (Figure 5). Based on their activities in apoptotic processes, expression of Bcl-2 and Bim was changed in a manner that would inhibit apoptosis in the NASH tissues. However, expression of another anti-apoptotic regulator, Mcl-1, was changed in a manner that would promote apoptosis. Thus, poorly controlled regulation of Bcl-2 family protein expression may contribute to dysregulation of apoptotic processes in NASH tissues.

Autophagy is a pathway mediating cell survival, although it can also promote cell death under certain conditions. Decreased autophagic function was reported to promote the initial development of NAFLD [47]. The observation that levels of p62 were unchanged suggests that the autophagy process was not activated in the NASH tissues (Figure 6), even though two major autophagy regulators, Atg16L1 and LC3, were induced.

NAFL may represent an intermediate state leading to NASH, the most extreme form of NAFLD. Five to twenty percent of patients with NAFL progress to NASH [48]. Levels of most of the proteins analyzed in NAFL tissues were either similar to those in one of the other two types, NASH and normal, or were somewhere in between (Figures 1, 2, 3, 4, 5, 6). That is, NAFL tissues showed no unique expression patterns of any of the proteins analyzed, as compared with NASH and normal liver tissues.

Taken together, our findings suggest that many proteins related to UPR, apoptosis and autophagy were dysregulated in the NASH tissues. Some of the proteins were dysregulated in NASH tissues in a manner consistent with inhibition of UPR and apoptosis processes. Inhibition of UPR and apoptosis can cause prolonged accumulation of cellular stresses that may, in turn, result in cell transformation. This is interesting to consider because NASH is one of the risk factors for hepatocellular carcinoma. Future studies are warranted to determine tissue environmental factors and signaling pathways that regulate expression of proteins related to UPR and apoptosis in NAFLD tissues.

## MATERIALS AND METHODS

### Chemicals

All chemicals used in this study were of reagent grade or higher and were purchased from Sigma-Aldrich (St. Louis, MO, USA), unless otherwise specified.

### Liver tissues

Frozen human liver tissues were obtained from the National Institutes of Health-funded Liver Tissue Cell Distribution System at the University of Minnesota, Virginia Commonwealth University and the University of Pittsburgh [49]. Patient IDs for the liver tissues used are listed in Supplementary Table 1. Clinical, histopathological, and donor information for the tissues was described previously [49].

### Tissue subcellular fractionation

Subcellular extraction of the liver tissues was performed as described by Cox and Emili [50]. Briefly, tissues were homogenized using a tight-fitting Teflon pestle in ice-cold lysis buffer containing 250 mM sucrose, 50 mM Tris-HCl (pH 7.4), 5 mM MgCl_2_, 1 mM dithiothreitol (DTT) and 1 mM phenylmethylsulfonyl fluoride (PMSF). The lysate was centrifuged at 6000 x g for 15 min at 4°C and the pellet resuspended to obtain mitochondrial proteins solubilized in extraction buffer containing 20 mM Tris-HCl (pH 7.8), 0.4 M NaCl, 15% glycerol, 1 mM DTT, 1 mM PMSF and 1.5% Triton-X-100. The supernatant was centrifuged at 100,000 x g for 1 h at 4°C, and the pellet resuspended to extract microsomal proteins into the extraction buffer. Protein concentrations were determined with a BCA protein assay kit (Pierce Biotechnology, Rockford, IL, USA).

### Western blot analysis

Sample preparation and western blotting were performed as previously described [51]. Anti-Atg5-Atg12 complex (#4180), -Atg16L1 (#8089), -Bak (#12105), -Beclin1 (#3495), -Bim (#2933), -Calnexin (#2679), -CHOP (#2895), -cleaved PARP (#9541), -cytochrome *c* (#4280), -Ero1-Lα (#3264), -ERp44 (#3798), -ERp72 (#5033), -GRP94 (#2104), -LC3A/B-I/II (#12741), -Mcl-1 (#5453), -PDI (#3501) and -VDAC (#4661) antibodies were from Cell Signaling Technology (Beverly, MA, USA). Anti-β-actin (ab8226), -Bcl-2 (ab692), -cleaved caspase-3 (ab2302), -GAPDH (ab9485), -GRP78 (ab21685) and -p62 (ab56416) antibodies were from Abcam (Cambridge, MA, USA). Anti-XBP1s (sc-7160) antibody was from Santa Cruz Biotechnology (Santa Cruz, CA, USA). Anti-cleaved ATF6 (NBP1-40256) antibody was from Novus Biologicals (Littleton, CO). Horseradish peroxidase-conjugated goat anti-mouse and anti-rabbit immunoglobulin G secondary antibodies were from GenDEPOT (Barker, TX, USA). The blots were developed using a chemiluminescent detection kit (Ab Frontier, Seoul, Republic of Korea). Densitometric quantification of western blot bands was performed using Image J software, version 1.49 (http://rsb.info.nih.gov/ij/index.html).

### Statistics

The nonparametric Mann-Whitney *U* test was performed to compare specific protein abundances in NASH and normal liver tissue groups (SAS 9.13 statistical program, SAS Institute, Cary, NC, USA). A *P*-value of < 0.05 was considered significant.

## SUPPLEMENTARY MATERIALS FIGURES AND TABLES



## Abbreviations

ATF6: activating transcription factor 6
Atg5: autophagy protein 5
Atg12: autophagy protein 12
Atg16L1: autophagy protein 16L1
Bak: bcl-2 homologous antagonist/killer
Bcl-2: B-cell lymphoma-2
Bim: bcl-2-like protein 11
CHOP: C/EBP homologous protein
DTT: dithiothreitol
ER: endoplasmic reticulum
Ero1: ER oxireductin 1
ERp44: ER protein 44
ERp72: ER protein 72
GAPDH: glyceraldeyde-3-phosphate dehydrogease
GRP78: glucose-regulated protein 78
GRP94: glucose-regulated protein 94
IRE1: inositol requiring enzyme 1
LC3A/B: microtubule-associated proteins 1A/1B light chain 3A
Mcl-1: myeloid cell leukemia-1
NAFL: nonalcoholic fatty liver
NAFLD: nonalcoholic fatty liver disease
NASH: nonalcoholic steatohepatitis
PARP: poly (ADP-ribose) polymerase
PDI: protein disulfide isomerase
PERK: protein kinase
PMSF: phenylmethylsulfonyl fluoride
RNA: like ER kinase
UPR: unfolded protein response
VDAC: voltage-dependent anion-selective channel
XBP1: X-box–binding protein 1
