# Supplementary material for: Dysregulated expression of proteins associated with ER stress, autophagy and apoptosis in tissues from nonalcoholic fatty liver disease
Source: Oncotarget. 2017 Jun 28;8(38):63370–81. doi: 10.18632/oncotarget.18812 (PMC5609929; doi:10.18632/oncotarget.18812)
Supplement: Supplementary file 1 [file oncotarget-08-63370-s001.pdf]

Dysregulated expression of proteins associated with ER stress, autophagy and apoptosis in tissues from nonalcoholic fatty liver disease

SUPPLEMENTARY MATERIALS

Supplementary Table 1: Clinical and donor information for the liver tissues used in this study

Sample #	Diagnosis	Patient ID	Gender	Age
Normal 1	Normal	D486	Female	58
Normal 2	Normal	D585	Male	60
Normal 3	Normal	D587	Male	49
Normal 4	Normal	UC9113	Female	59
NAFL 1	NAFL	D218	Female	51
NAFL 2	NAFL	UMN1058	Unknown	Unknown
NASH 1	NASH	D346	Female	55
NASH 2	NASH	D515	Female	56
NASH 3	NASH	UMN935	Female	60
